# Supplementary material for: High-intensity UV laser ChIP-seq for the study of protein-DNA interactions in living cells
Source: Nat Commun. 2017 Nov 3;8:1303. doi: 10.1038/s41467-017-01251-7 (PMC5670203; doi:10.1038/s41467-017-01251-7)
Supplement: Supplementary file 1 — Supplementary Information [file 41467_2017_1251_MOESM1_ESM.pdf]

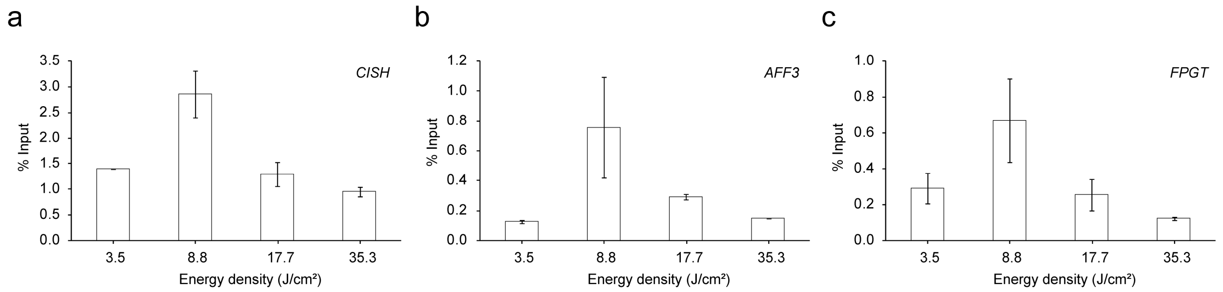

Supplementary Figure 1. UV dose titration by BCL6 UV-ChIP-qPCR. The optimum UV dose obtaining maximum enrichment of photochemical crosslinked BCL6-DNA complexes in OCI-LY1 cells was identified by UV-ChIP-qPCR of BCL6 binding sites within the promoter region of the (a) *CISH*, (b) *AFF3* and (c) *FPGT* human genes (Supplementary Table 1)[1–3]. The total energy density for UV dose titration ranged 3.5 to 35.3 J cm^{-2}. Experimental values represent the mean percentage of input (% Input) for enrichment of BCL6 at sequence-specific binding sites. The enrichment of BCL6 within the UV IgG control sample (% Input <0.06) and of BCL6 at specific control regions (% Input <0.02) is not shown within the figure. Data represent the mean of at least two determinations in triplicate from each of two independent UV-ChIP experiments (a minimum of four determinations, error bars represent s.d.). UV laser irradiation of cells, subsequent ChIP-qPCR experimentation and data analysis was performed according Methods.

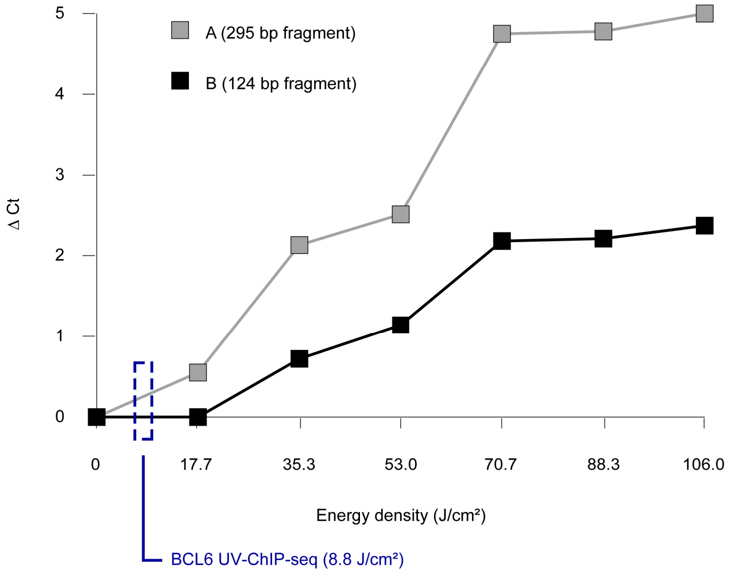

Supplementary Figure 2. Effect of UV laser irradiation on amplificability of DNA. DNA damage was estimated following high-intensity nano-second UV laser irradiation (energy density 0 to 106 J cm^{-2}) of OCI-LY1 cells, DNA isolation and subsequent qPCR analysis of two genomic regions A (295 bp) and B (124 bp). Ct values were normalized to input DNA (non-irradiated control, -UV), delta Ct values (ΔCt = Ct [UV] – Ct [-UV]) were calculated and plotted. Final BCL6 UV-ChIP-seq experiments were performed at a UV dose of 8.8 J cm^{-2} as obtained by UV dose titration for photochemical crosslinking of BCL6 in OCI-LY1 cells (Supplementary Fig. 1).

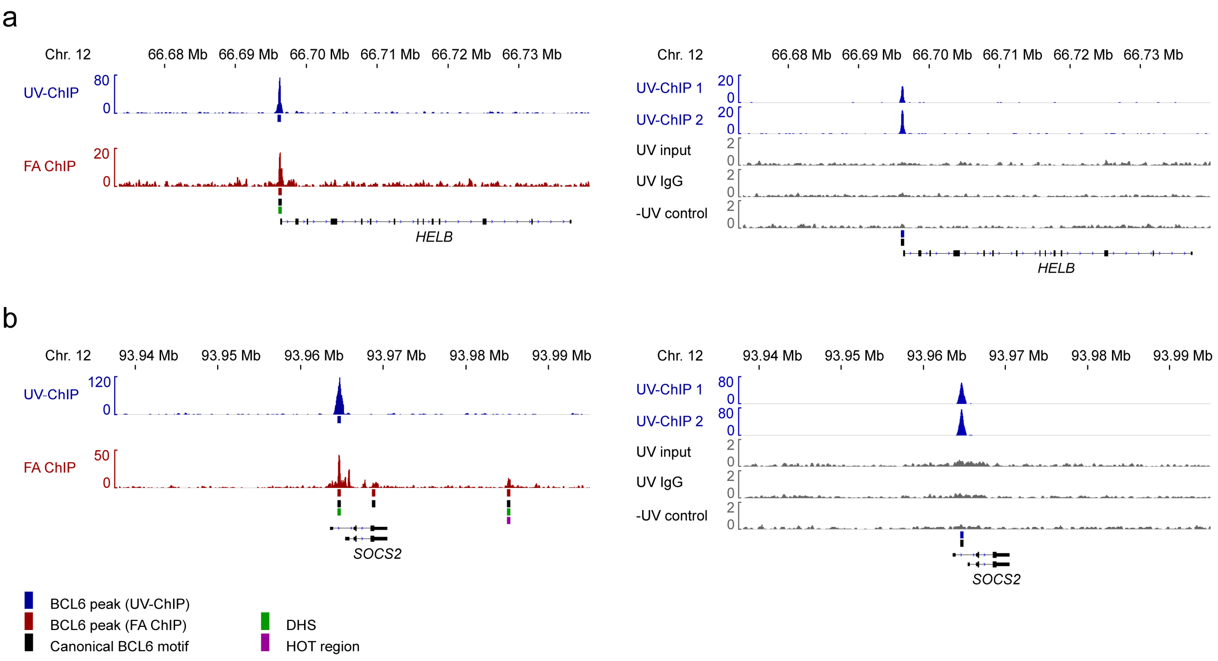

Supplementary Figure 3. Binding profiles of direct BCL6-DNA interactions. Examples of previously validated BCL6 interaction sites within the promoter region of the human (a) *HELB* and (b) *SOCS2* genes[1,4]. UV-ChIP-seq (UV-ChIP, blue) and FA ChIP-seq (FA ChIP, red) read density profiles exhibit specific enrichment at corresponding canonical BCL6 binding motifs within accessible chromatin regions (DHS/HOT region). Additional non-overlapping binding sites exist for the (b) *SOCS2* genomic loci. FA ChIP-seq shows enrichment intragenic (last exon) and around 14.5 kb downstream the *SOCS2* gene. Comparative profiles (left) are shown as fold enrichment track generated by ChIP over input DNA pileup signal (normalized) with corresponding scales indicated. Individual UV read density profiles (right) are shown as pileup signal track normalized by sequencing depth. Note the different scales in UV-ChIP and UV control profiles. Genomic coordinates and human RefSeq annotations (GRCh37/hg19) are shown. Blue (UV-ChIP) and red (FA ChIP) boxes correspond to detected BCL6 binding sites. Black boxes indicate canonical BCL6 binding motif appearance (q-value 0.1) and peaks overlapping DHSs (green boxes) and HOT regions (purple boxes) are marked.

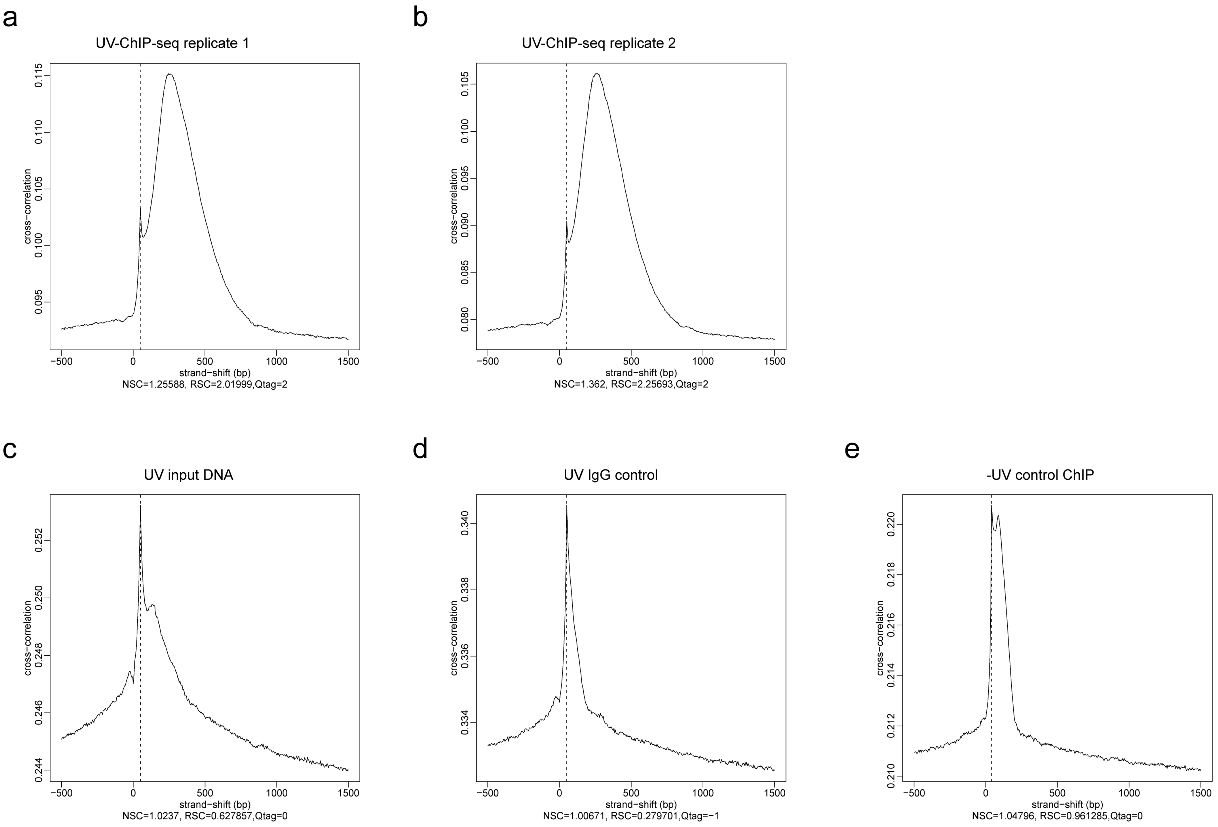

Supplementary Figure 4. Strand cross-correlation (SCC) analysis. SCC analysis was performed to assess signal-to-noise ratios in UV-ChIP and UV control (UV input DNA, UV IgG control and -UV control ChIP) sequencing data. Cross-correlation plot profiles and corresponding metrics (NSC, RSC, Qtag) are shown for (a,b) biological replicate BCL6 UV-ChIP-seq, (c) UV input DNA, (d) UV IgG control and (e) -UV control ChIP data. Dashed lines indicate the strand shift, which correspond to the sequenced read length (50 bp). NSC, RSC and Qtag metrics are shown and summarized in Supplementary Table 2.

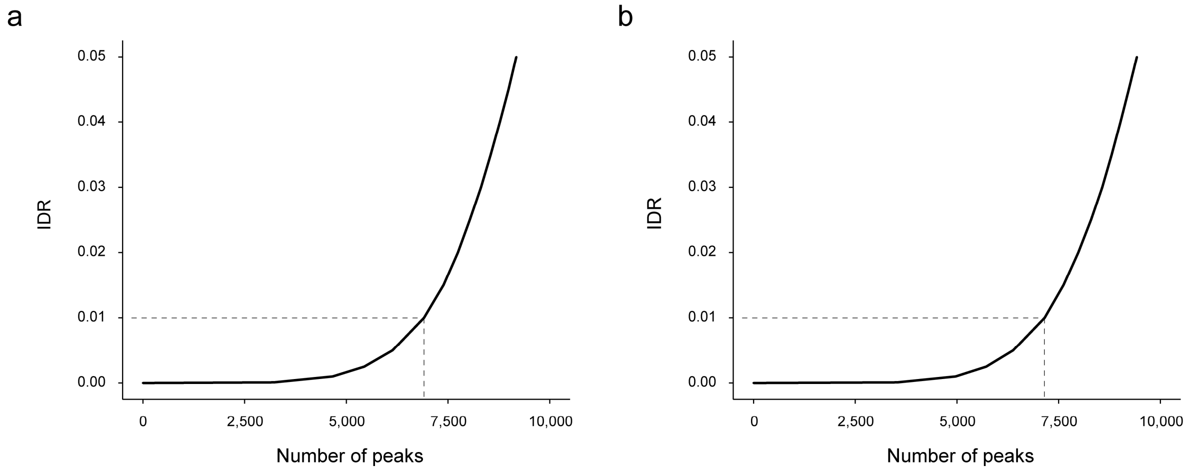

Supplementary Figure 5. Irreproducible Discovery Rate (IDR) framework. The IDR framework was performed for assessing the reproducibility and for peak calling in BCL6 UV-ChIP-seq (a) replicate and (b) pseudo-replicate (pooled and subsampled reads from replicates) datasets. The estimated IDR at different rank thresholds was plotted and the number of final peaks (replicate dataset n=6,910; pseudo-replicate dataset n=7,156) at an IDR of 0.01 is indicated (dashed line) (Supplementary Table 3).

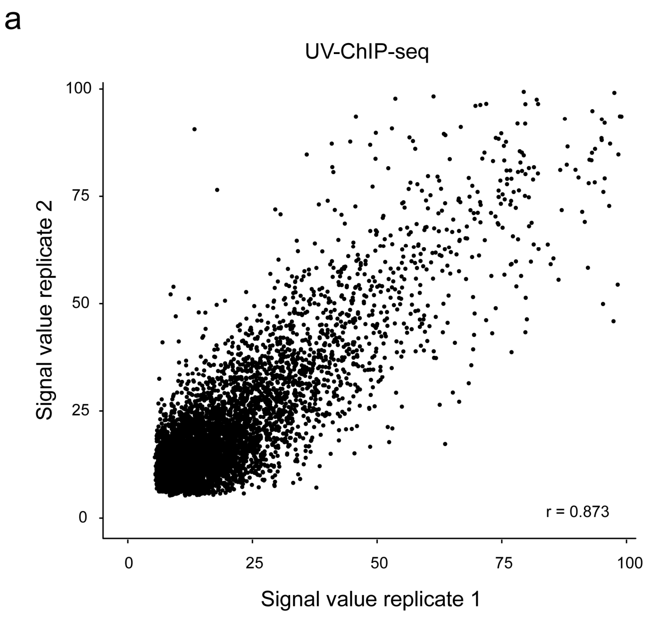

Supplementary Figure 6. Comparative peak calling by the IDR algorithm. (a) Peak detection in individual BCL6 UV-ChIP-seq replicate experiments by the IDR framework. Each point represent the signal values of overlapping peaks (IDR 0.01, n=6,910) from UV-ChIP-seq replicate 1 and 2. The Pearson correlation (r) is indicated.

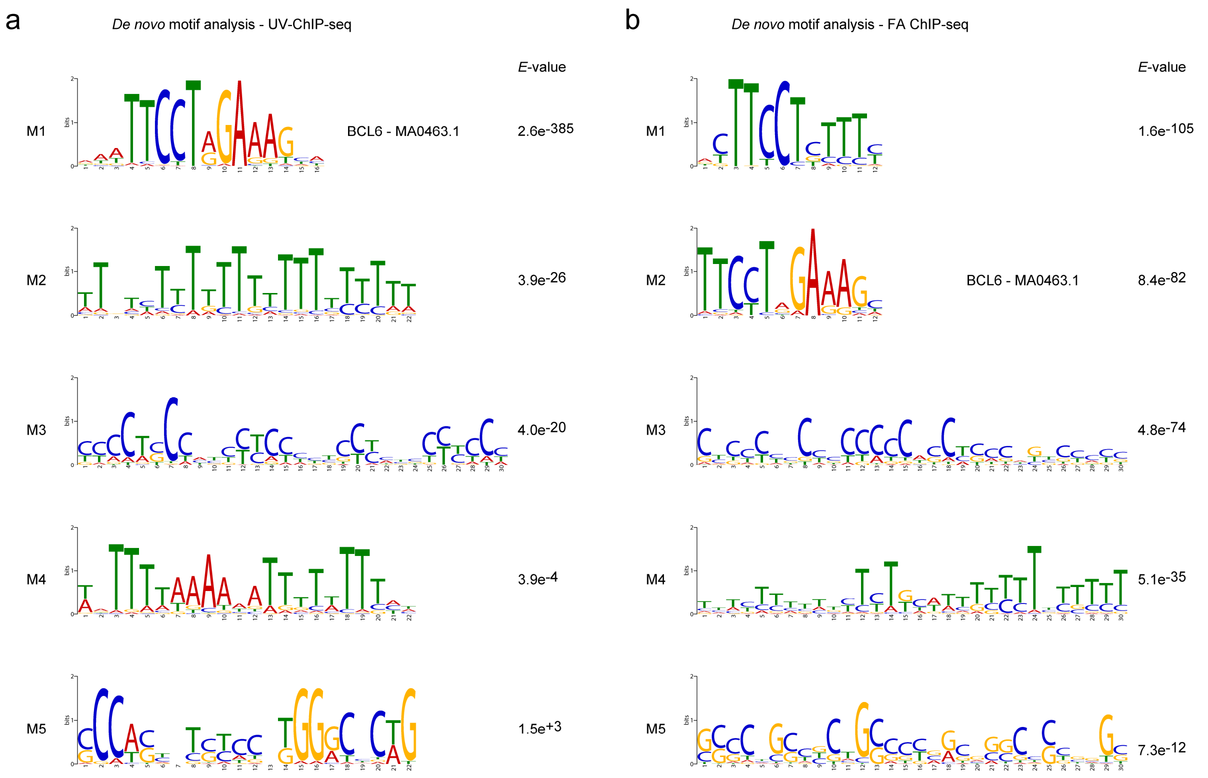

Supplementary Figure 7. *De novo* motif analysis. *De novo* motif analysis (5 motifs; M1 – M5) within top ranked detected BCL6 binding sites (n=500) from (a) UV-ChIP-seq and (b) FA ChIP-seq data analyses was performed and corresponding sequence logos and *E*-values are shown. Motif comparison against the JASPAR CORE database showed that *de novo* motif one (M1) in UV-ChIP-seq and motif two (M2) in FA ChIP-seq data correspond to the canonical BCL6 binding motif (MA0463.1) previously reported[5].

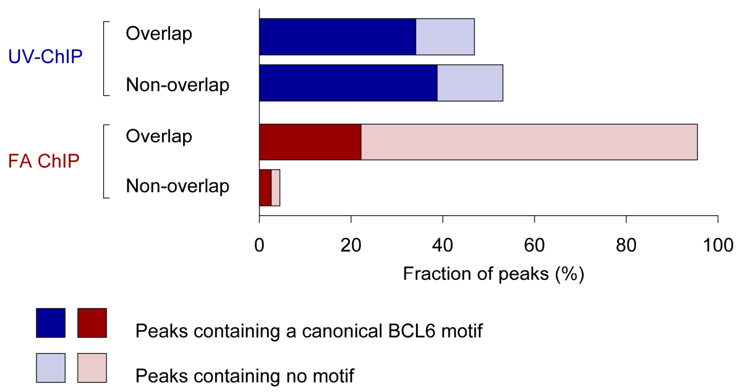

Supplementary Figure 8. BCL6 binding relative to known DHSs. The fractions (overlap, non-overlap) of identified BCL6 binding sites by UV-ChIP-seq (blue) and FA ChIP-seq (red) relative to the ENCODE DNaseI Hypersensitive Site (DHS) Master List (DHSs detected in 127 cell types, 2.9 mil DHSs) are shown[6]. The proportion of peaks containing a canonical BCL6 motif (*q*-value 0.1) is indicated (dark colored).

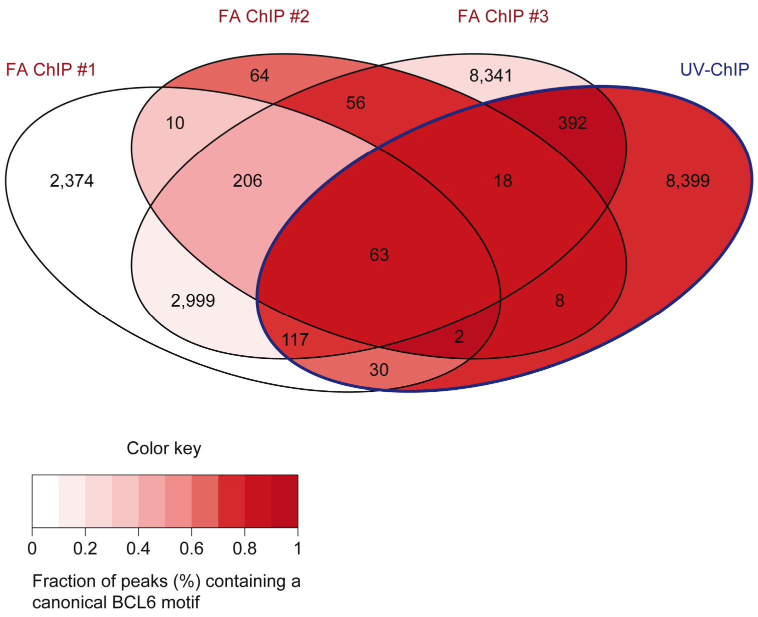

Supplementary Figure 9. BCL6 binding site overlap. The individual overlap of BCL6 binding sites identified in OCI-LY1 cells by UV-ChIP-seq and FA ChIP-seq (FA ChIP #1 GSE29282, FA ChIP #2 GSE103125 and FA ChIP #3 GSE46663) data analyses is summarized by a venn diagram[7,8]. Numbers indicate overlapping BCL6 peaks and the fraction of peaks (%) containing a canonical BCL6 motif (*q*-value 0.1) is illustrated (see color key).

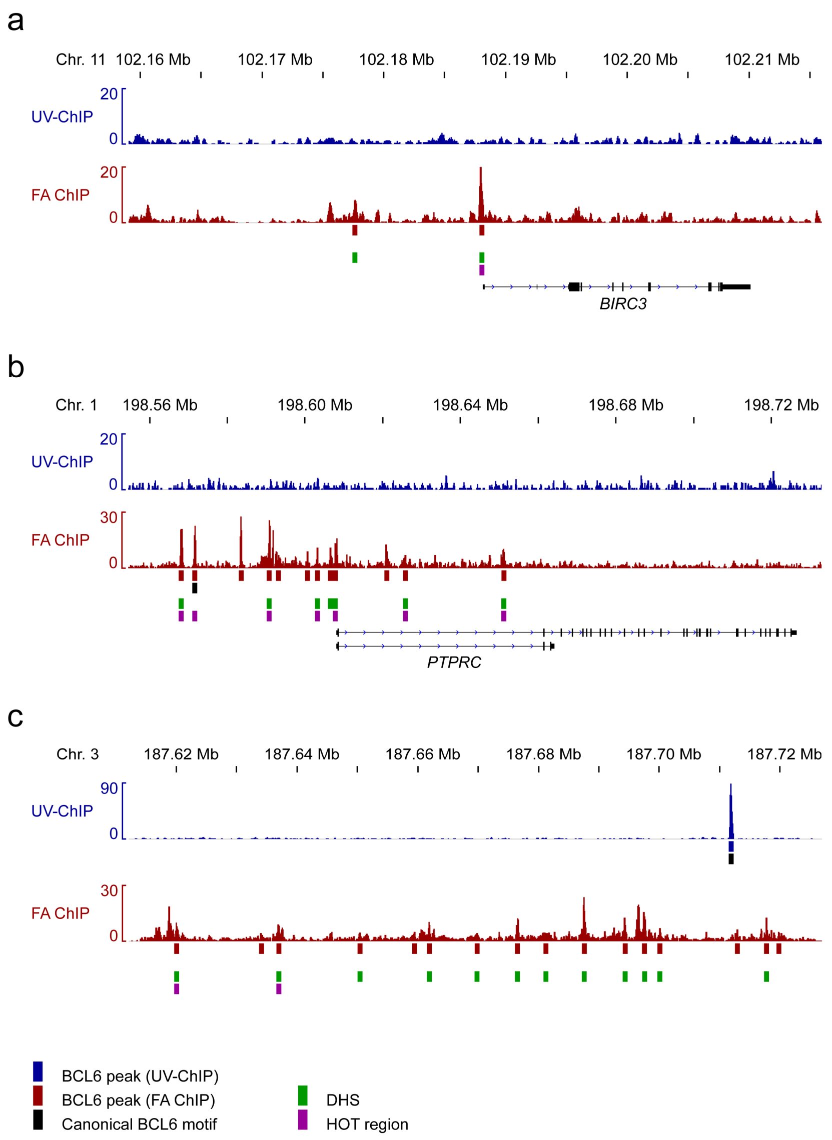

Supplementary Figure 10. Non-overlapping BCL6 binding profiles. Examples of UV-ChIP-seq (UV-ChIP, blue) and FA ChIP-seq (FA ChIP, red) profiles are shown for (a) *BIRC3*, (b) *PTPRC* human genomic loci and the (c) *BCL6* upstream enhancer region[8]. In contrast to UV-ChIP-seq, various BCL6 binding sites to non-canonical DNA sequence motifs were detected by FA ChIP-seq within accessible chromatin regions (DHS and HOT region). The (c) *BCL6* upstream enhancer region exhibit multiple BCL6 binding sites to non-canonical DNA motifs detected by FA ChIP-seq. One single BCL6 binding site overlapping a canonical BCL6 motif was detected by UV-ChIP-seq. Read density profiles are shown as fold enrichment track generated by ChIP over input DNA pileup signal (normalized) with the corresponding scales as indicated. Genomic coordinates and human RefSeq annotations (GRCh37/hg19) are shown. Blue (UV-ChIP) and red (FA ChIP) boxes correspond to detected BCL6 binding sites. Black boxes indicate canonical BCL6 motif appearance (*q*-value 0.1) and peaks overlapping DHSs (green boxes) and HOT regions (purple boxes) are marked.

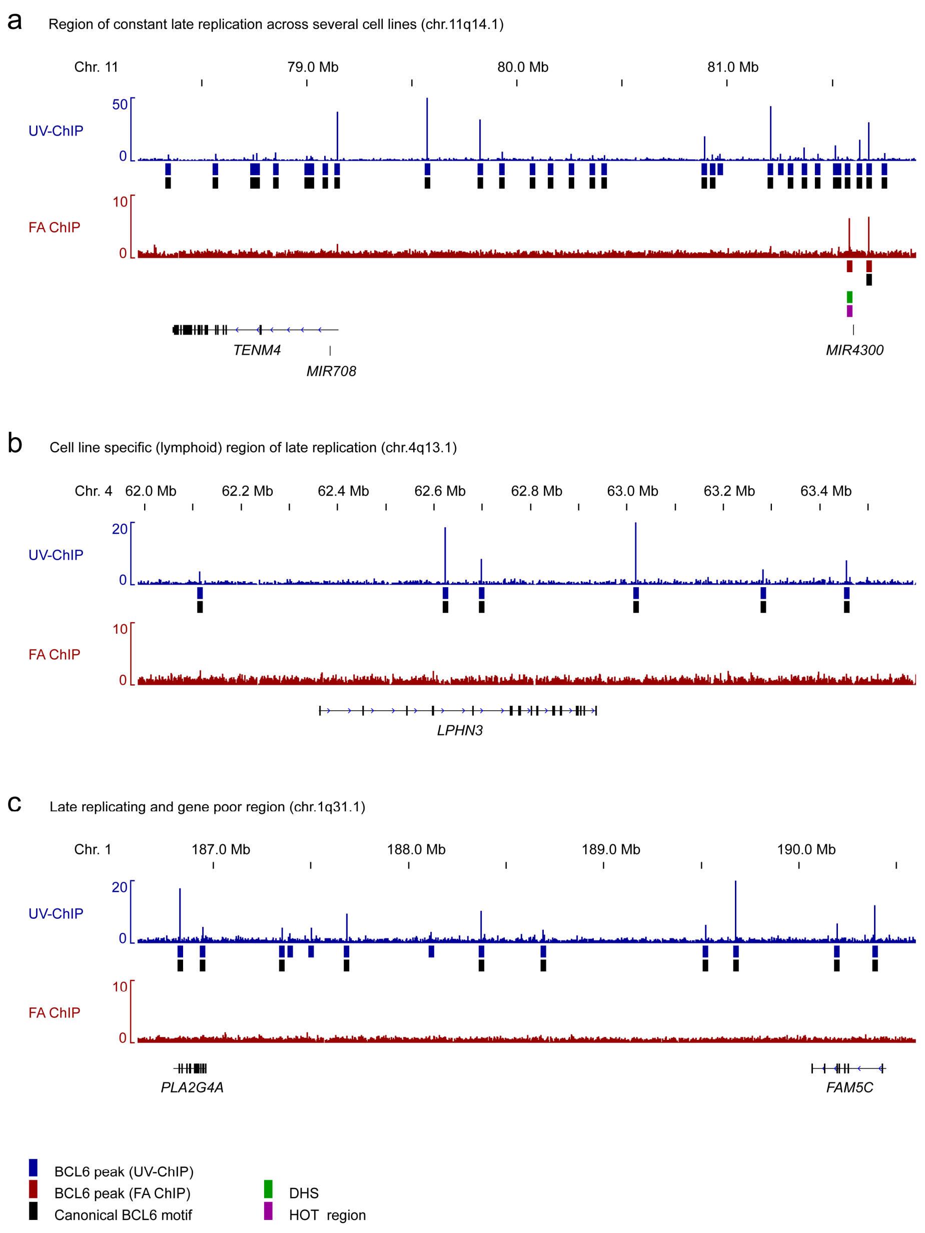

Supplementary Figure 11. BCL6 binding within inaccessible chromatin. Examples of UV-ChIP-seq (UV-ChIP, blue) and FA ChIP-seq (FA ChIP, red) profiling within late replicating and gene poor human genomic regions at (a) chr. 11q14.1, (b) chr. 4q13.1, and (c) chr. 1q31.1[9–11]. In summary, UV-ChIP-seq proved to be sensitive and specific in the detection of BCL6 binding sites to corresponding canonical DNA motifs within these inaccessible chromatin domains, which were not detected by FA ChIP-seq. Read density profiles are shown as fold enrichment track generated by ChIP over input DNA pileup signal (normalized) with the corresponding scales as indicated. Genomic coordinates and human RefSeq annotations (GRCh37/hg19) are shown. Blue (UV-ChIP) and red (FA ChIP) boxes correspond to detected BCL6 binding sites. Black boxes indicate canonical BCL6 motif appearance (*q*-value 0.1) and peaks overlapping DHSs (green boxes) and HOT regions (purple boxes) are marked.

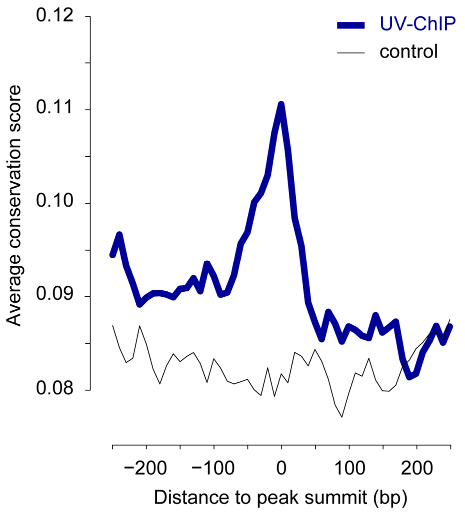

Supplementary Figure 12. BCL6 binding site conservation. The average conservation score of DNA sequences bound by BCL6 (blue) is shown relative to the peak summit (base position of maximum enrichment, x axis, ± 250 bp) of newly identified sequence-specific BCL6 binding sites (n=6,026) by UV-ChIP-seq data analyses and compared to random sequences (control, grey).

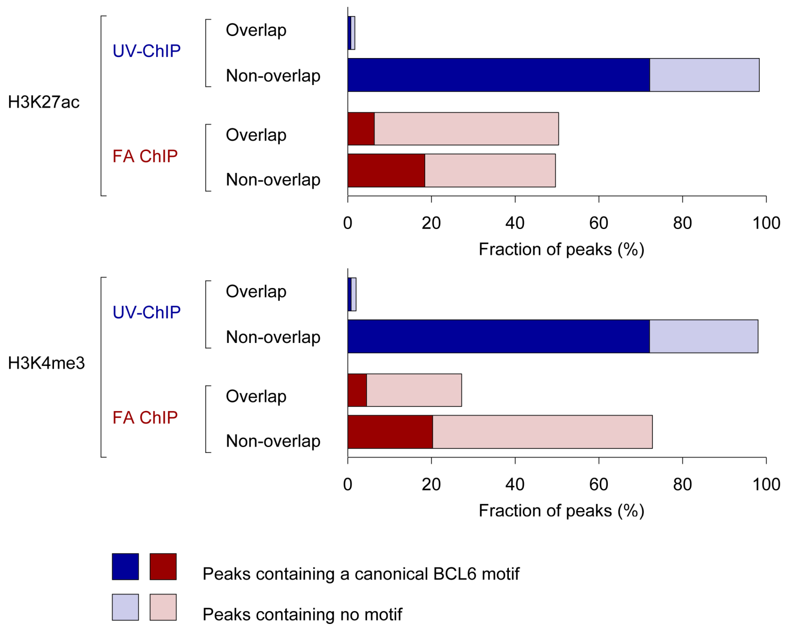

Supplementary Figure 13. BCL6 binding relative to histone marks. The fractions (overlap, non-overlap) of BCL6 binding sites (%) identified by UV-ChIP-seq (blue) and FA ChIP-seq (red) relative to the activating histone marks H3K27ac (GSM763424) and H3K4me3 (GSM763420) in human OCI-LY1 cells are shown[7]. The proportion of peaks containing a canonical BCL6 motif (*q*-value 0.1) is indicated (dark colored).

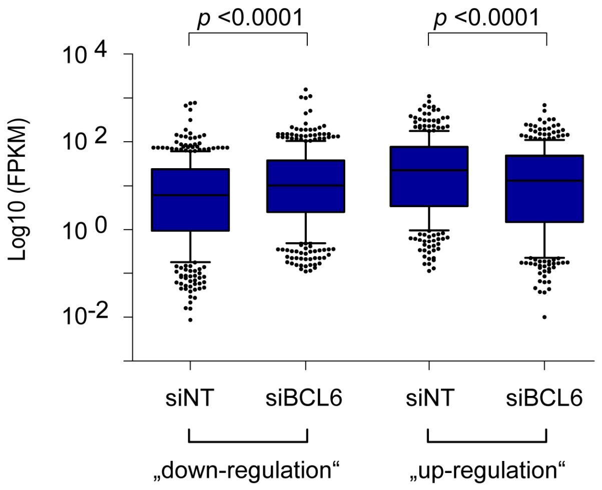

Supplementary Figure 14. Differential gene expression analysis. The expression status of differentially expressed genes (*q*-value 0.05) was analyzed following knockdown of BCL6 (siBCL6) compared to control data (siNT) in OCI-LY1 cells. The distribution of paired gene expression values (Log10 FPKM, fragments per kilobase of transcript per million mapped reads) of genes associated with BCL6 binding sites identified by UV-ChIP-seq is shown by box plots (black line represent the median and whiskers the 10-90th percentile). Paired expression values (siNT, siBCL6) were plotted for each group of genes which show differential down-regulation (n=413) or up-regulation (n=334) potentially by BCL6 (Supplementary Data 2).

Locus			Sequence 5'-3'
AFF3	+	For	GAA CAA AGG GGA GAG TGC TG
		Rev	AGT TGG CCT AGA ACC GGA AG
	Δ 2.4 kb	For	AGA GGG AAA GGC CTG AAG AC
		Rev	CCA TCT GCT TCT CCT TCC AG
CISH	+	For	TCC TGG AAA GTT CTT GGA AAT C
		Rev	AGC TGC TGC CTA ATC CTT TG
	Δ 3.2 kb	For	CTG GAC CCA GGA TTG ATG TC
		Rev	CTG CCT AGC CTT CTG CTC AC
FPGT	+	For	CAC TAT TTG AGA GGC TGT CGT G
		Rev	CTG GGT CAT GAT ACA TAC ACA GTC
	Δ 2.5 kb	For	GTT CCT CCA ACA GGG CAT AG
		Rev	AGC TCG TAG TGC ATG CTG TG
A		For	TCA CCC ACA CTG TGC CCA TCT ACG A
		Rev	TCA CCC ACA CTG TGC CCA TCT ACG A
B		For	GAC ACC CCA CGC CAG TT
		Rev	GAT CCC CAT TGG CAA GA

Supplementary Table 1. Primer sequences used for UV-ChIP-qPCR

Experiment	Raw reads	Uniquely mapped reads		Non-redundant reads	NSC	RSC	QC tag
UV-ChIP-seq Repl. 1	29,768,127	21,376,069	71.8 %	7,105,236	1.26	2.02	2
UV-ChIP-seq Repl. 2	29,601,539	21,594,517	73.0 %	5,919,371	1.36	2.26	2
UV Input DNA	27,292,215	22,609,605	82.8 %	22,218,603	1.02	0.63	0
UV IgG control	52,860,113	37,516,689	71.0 %	35,069,375	1.01	0.28	-1
–UV control ChIP	49,308,218	30,038,627	60.9 %	18,649,294	1.05	0.96	0
FA ChIP-seq	103,079,340	80,122,490	75.8 %	30,231,758			
FA Input DNA	37,759,984	30,884,965	81.8 %	30,250,727			
FA IgG control	79,653,661	62,514,037	78.5 %	46,947,063			

Supplementary Table 2. ChIP sequencing data

Experiment	IDR analysis	IDR threshold	Peaks (ChIP/IgG)	Peaks (ChIP/Input)	
UV-ChIP-seq Repl. 1/2	replicates	0.01	6,918	6,910	(N_t)
UV-ChIP-seq (pooled)	pseudo-replicates	0.01	6,982	7,156	(N_p)
				N_p/N_t=1.04	
UV-ChIP-seq Repl. 1	pseudo-replicates	0.01	3,234	3,320	(N_1)
UV-ChIP-seq Repl. 2	pseudo-replicates	0.01	2,989	2,991	(N_2)
				N_1/N_2=1.11	

Supplementary Table 3. Irreproducible Discovery Rate (IDR) framework

Supplementary References

1. Chen, Y. *et al.* Computational and functional analysis of growth hormone-regulated genes identifies the transcriptional repressor Bcl6 as a participant in GH-regulated transcription. *Endocrinology* (2009). doi:10.1210/en.2009-0212
2. Ci, W. *et al.* The BCL6 transcriptional program features repression of multiple oncogenes in primary B cells and is deregulated in DLBCL. *Blood* **113,** 5536–5548 (2009).
3. Basso, K. *et al.* Integrated biochemical and computational approach identifies BCL6 direct target genes controlling multiple pathways in normal germinal center B cells. *Blood* **115,** 975–984 (2010).
4. Polo, J. M. *et al.* Transcriptional signature with differential expression of BCL6 target genes accurately identifies BCL6-dependent diffuse large B cell lymphomas. *Proc. Natl. Acad. Sci. U. S. A.* **104,** 3207–3212 (2007).
5. Mathelier, A. *et al.* JASPAR 2014: an extensively expanded and updated open-access database of transcription factor binding profiles. *Nucleic Acids Res.* **42,** D142–7 (2014).
6. Thurman, R. E. *et al.* The accessible chromatin landscape of the human genome. *Nature* **489,** 75–82 (2012).
7. Hatzi, K. *et al.* A hybrid mechanism of action for BCL6 in B cells defined by formation of functionally distinct complexes at enhancers and promoters. *Cell Rep.* **4,** 578–588 (2013).
8. Chapuy, B. *et al.* Discovery and characterization of super-enhancer-associated dependencies in diffuse large B cell lymphoma. *Cancer Cell* **24,** 777–790 (2013).
9. Hansen, R. S. *et al.* Sequencing newly replicated DNA reveals widespread plasticity in human replication timing. *Proc. Natl. Acad. Sci. U. S. A.* **107,** 139–144 (2010).
10. Ryba, T. *et al.* Evolutionarily conserved replication timing profiles predict long-range chromatin interactions and distinguish closely related cell types. *Genome Res.* **20,** 761–770 (2010).
11. Julienne, H., Zoufir, A., Audit, B. & Arneodo, A. Human Genome Replication Proceeds through Four Chromatin States. *PLoS Comput. Biol.* **9,** (2013).
