# Supplementary material for: High-intensity UV laser ChIP-seq for the study of protein-DNA interactions in living cells
Source: Nat Commun. 2017 Nov 3;8:1303. doi: 10.1038/s41467-017-01251-7 (PMC5670203; doi:10.1038/s41467-017-01251-7)
Supplement: Supplementary file 3 — Description of Additional Supplementary Files [file 41467_2017_1251_MOESM3_ESM.pdf]

File Name: Supplementary Data 1
Description: Potential BCL6 target genes. Target gene prediction based on identified BCL6 binding sites by UV-ChIP-seq in human DLBCL cells. Gene symbol, peak distances from the transcription start site (TSS), ensemble gene id and gene description is provided.

File Name: Supplementary Data 2
Description: Differentially expressed potential BCL6 target genes. List of genes differentially expressed following knockdown of BCL6 in OCI-LY1 cells found to have BCL6 binding sites in their regulatory regions. Gene symbol, peak distances from the transcription start site (TSS), ensemble gene id and gene description is provided. Putative new target genes exclusively found by UV-ChIP-seq are marked (new target).
